# Toxicity of nickel ions and comprehensive analysis of nickel ion-associated gene expression profiles in THP-1 cells

**DOI:** 10.3892/mmr.2015.3878

**Published:** 2015-06-03

**Authors:** YING ZHANG, ZHI-WEI ZHANG, YU-MEI XIE, SHU-SHUI WANG, QING-HUAN QIU, YING-LING ZHOU, GUO-HONG ZENG

**Affiliations:** 1 Departments of Cardiology, Guangdong Provincial Cardiovascular Institute, Guangdong General Hospital, Guangdong Academy of Medical Sciences, Guangzhou, Guangdong 510180, P.R. China; 2 Departments of Pediatric Cardiology, Guangdong Provincial Cardiovascular Institute, Guangdong General Hospital, Guangdong Academy of Medical Sciences, Guangzhou, Guangdong 510180, P.R. China

**Keywords:** toxicity, nickel ion, THP-1 cell, Illumina sequencing

## Abstract

The aim of the present study was to explore the toxic effects and underlying mechanisms of nickel ions during therapeutic nickel-based alloy-treatment in congenital heart disease by investigating the metal-induced cytotoxicity to the human monocyte-derived macrophage cell line THP-1. THP-1 cells were treated with NiCl_2_·6H_2_O (25, 50, 100, 200, 400 and 800 *µ*M) for 24, 48 and 72 h, respectively. MTT was applied to detect THP-1 cell proliferation following NiCl_2_ treatment. Apoptosis of THP-1 cells was quantified using flow cytometry. Illumina sequencing was used for screening the associated genes, whose mRNA expression levels were further confirmed by quantitative real-time polymerase chain reaction. High concentrations of nickel ions had a significant suppressive effect on cell proliferation at the three concentrations investigated (200, 400 and 800 *µ*M). Treatment with nickel ions (25–400 *µ*M) for 48 h reduced cell viability in a dose-dependent manner. The mRNA expression levels of RELB, FIGF, SPI-1, CXCL16 and CRLF2 were significantly increased following nickel treatment. The results of the present study suggested that nickel ions exert toxic effects on THP-1 cell growth, which may indicate toxicity of the nickel ion during treatment of congenital heart disease. The identification of genes modified by the toxic effects of nickel on THP-1 cells (EPOR, RELB, FIGF, SPI-1, TGF-β1, CXCL16 and CRLF2) may aid in the development of interventional measures for the treatment/prevention of nickel ion-associated toxic effects during the treatment of congenital heart disease.

## Introduction

Congenital heart disease is the most common type of congenital malformation (1). Only certain types of congenital heart disease are able to recover naturally, and others present with increased severity and frequency of complications with increasing age (2). Interventional therapy is one of the therapeutic methods used for congenital heart disease (2). Blocking cardiac defects with metal occluders of different shapes is a commonly used surgical alternative, with the nickel-chromium (Ni-Cr) alloy occluder being most widely used in the clinical setting (3).

The clinical application of the Ni-Cr alloy occluder is a result of advances in medical technology and material sciences, and that of a process of continuous research and improvement. Use of the Ni-Cr alloy occluder avoids the risks and trauma of open-heart surgery, allowing short hospital stays and fast recovery for patients (4). However, long-term nickel-based alloy implantation has been increasingly documented to be associated with biological effects, including cytotoxicity and genotoxicity, eliciting tissue inflammation and potential sensitization, thus raising questions over its bio-safety (5). In previous experiments following-up children receiving an atrial septal defect occluder implantation, it was observed that the nickel concentration in the blood was significantly increased 24 h and one month subsequent to surgery (Zhang *et al*; unpublished results). Other studies have also reported that the Ni-Cr alloy occluder implantation results in the release of the nickel ion into the chambers of the heart, which may result in cell damage due to local and systemic toxicity (6) and lead to headaches, dyspnea and other unpleasant symptoms (7,8).

Being the 'first line of defense', macrophages are essential determinants of implant biocompatibility (9). Pro-inflammatory studies have observed that during the first six weeks following implantation, macrophages interact heavily with the prosthetic device (10). Therefore, the present study aimed to investigate the cytotoxicity of nickel ions on THP-1, a human monocytic cell line derived from the peripheral blood, and to explore the underlying mechanism of the toxicity of the nickel alloy in the heart during its application for congenital heart disease. The present study additionally aimed to provide further insights for physicians and patients on the clinical use and adverse effects of nickel alloy implantation in congenital heart disease and various additional associated diseases.

## Materials and methods

### Materials

The human monoblastic leukemia cell line THP-1 was obtained from the American Type Culture Collection (Manassas, VA, USA). Cell culture reagents RPMI 1640 medium and fetal bovine serum (FBS) were from Gibco-BRL (Invitrogen Life Technologies, Carlsbad, CA, USA). NiCl_2_·6H_2_O, MTT, Hoechst 33342 and propidium iodide (PI) were obtained from Sigma-Aldrich (St. Louis, MO, USA). TRIzol reagent was obtained from Invitrogen Life Technologies. All reagents were of analytical grade.

### Cell culture and drug treatment

THP-1 cells were cultured in RPMI 1640 medium supplemented with 10% FBS in a humidified atmosphere of 5% CO_2_ and 95% air at 37°C. Cells were serum-starved for 48 h prior to treatment with NiCl_2_·6H_2_O (25, 50, 100, 200, 400 or 800 *µ*M) for 24, 48 or 72 h, respectively.

### Cell growth assay

The MTT assay and cell-counting method were applied to detect THP-1 cell proliferation subsequent to receiving different treatment regimens. The apoptosis of THP-1 in response to nickel ion challenge was detected using flow cytometry (FCM). For the MTT assay, THP-1 cells were seeded in 96-well plates at a density of 4×10^5^ cells/well and then exposed to various concentrations of NiCl_2_·6H_2_O for the different time periods indicated (25, 50, 100, 200, 400 and 800 *µ*M for 24, 48 and 72 h). At the end of the treatments, the plates were centrifuged at 2,000 × g for 5 min and the supernatants were discarded. Then cells were further incubated with RPMI 1640 (200 *µ*l/well) supplemented with 0.2% MTT (20 *µ*l/well) for 4 h at 37°C. Following MTT incubation, 100 *µ*l 100% dimethyl sulfoxide was added to dissolve the formazan crystals. Viable cells were counted by reading the absorbance at 570 nm using a 96-well plate reader (Multiskan MK3; Thermo Fisher Scientific, Waltham, MA, USA).

### Cytotoxicity analysis

Cells were plated on six-well plates and were allowed to adhere. Following different treatments for 48 h, cells from each group (5×10^5^ cells/group re-suspended in 0.5 ml ice-cold RPMI 1640) were transferred into a clean centrifuge tube and incubated with 1.25 *µ*l Hoechst 33342 for 15 min at room temperature in the dark to stain the nuclei of all cells. Subsequently, the plates were centrifuged at 2,000 × g for 5 min and the supernatants were discarded. Cells were then re-suspended in 0.5 ml ice-cold RPMI 1640 prior to the addition of 10 *µ*l PI (5 *µ*g/ml) for staining of the dead cells. The samples were kept on ice in the dark and immediately analyzed using a FACSCalibur Cell flow cytometer (BD Biosciences, Franklin Lakes, NJ, USA). Cytotoxicity was expressed as the percentage of dead cells (PI-positive) relative to the total number of cells.

### RNA-sequencing assay

THP-1 cells were cultured with different concentrations of NiCl_2_·6H_2_O (25, 50 or 100 *µ*M) for 1 h following starvation for 48 h. Total RNA was prepared by the acid phenol method using TRIzol reagent in accordance with the manufacturer's instructions. Areas containing THP-1 RNAs were sent to Beijing Genomics Institution (Shenzhen, China) for Illumina sequencing.

### Pathway analysis of differentially expressed genes

Pathways were constructed using the Kyoto Encyclopedia of Genes and Genomes (KEGG; http://www.genome.jp/kegg/) database using the Web-based GEne SeT Analysis Toolkit (http://bioinfo.vanderbilt.edu/webgestalt/) to identify differential networks and core regulators involved in the nickel-induced cytotoxic response. The analysis completed was limited to categories with P<0.05.

### Quantitative polymerase chain reaction (qPCR)

PCR was performed using the total RNA (1 *µ*g) prepared for the RNA-sequencing assay with the primers listed in Table I under the following conditions: 40 cycles of 94°C for 2 min, 94°C for 30 sec, 58°C for 30 sec and 72°C for 20 sec. RNA expression levels were detected by fluorescent qPCR in the presence of SYBR Green on a Bio-Rad iCycler (Bio-Rad Laboratories, Inc., Hercules, CA, USA).

### Statistical analysis

Values are expressed as the mean ± standard deviation. The independent-samples t-test was used to compare two samples and one-way analysis of variance was used for multiple comparisons. P<0.05 was considered to indicate a statistically significant difference.

## Results

### Nickel ions inhibit THP-1 cell growth

**Figure 1:**
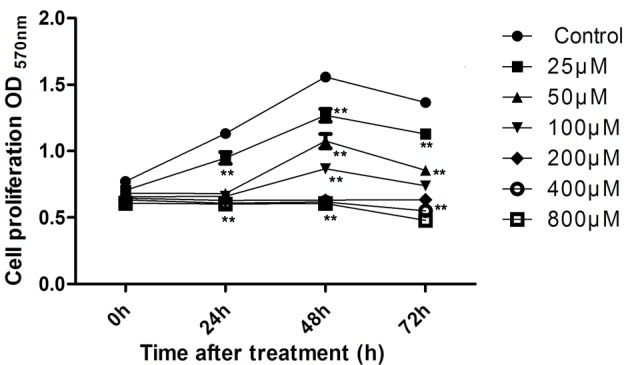
Effect of nickel ion treatment on cell proliferation. The OD of MTT-formazan was determined to assess cell proliferation at 0, 24, 48 and 72 h after treatment with NiCl_2_·6H_2_O at concentrations of 25, 50, 100, 200, 400 and 800 *µ*M (^**^P<0.001, compared with the control). OD, optical density.

The effect of nickel ions on the proliferation of THP-1 was investigated. Cells were treated with NiCl_2_·6H_2_O (25, 50, 100, 200, 400 and 800 *µ*M) for 24, 48 and 72 h, and an MTT assay was conducted in order to examine the growth-inhibitory effect of the treatments. As presented in Fig. 1, high concentrations of nickel chloride significantly suppressed cell proliferation at the three concentrations used (200, 400 and 800 *µ*M; P<0.001). Cell proliferation was completely abolished following incubation of cells with nickel chloride at these high concentrations. Lower concentrations of NiCl_2_·6H_2_O (25–100 *µ*M) inhibited cell proliferation in a time- and dose-dependent manner as compared with that in the control group.

### Nickel ions exert toxic effects on THP-1 cells

**Figure 2:**
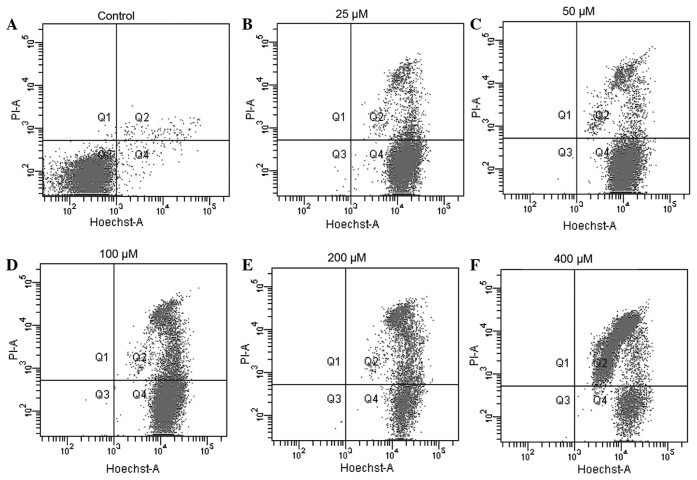
Analysis of cytotoxicity of nickel ions by flow cytometry. (A) THP-1 cells were treated with (B) 25, (C) 50, (D) 100, (E) 200 and (F) 400 *μ*M nickel ions and were harvested following 48-h treatment. Cytotoxicity is indicated by the percentage of dead cells (PI-positive) relative to the total number of cells. PI, propidium iodide.

To investigate the toxicity of the nickel ion on THP-1 cells, the viability of THP-1 cells treated with nickel chloride was assessed via FCM. As illustrated in Fig. 2, treatment with nickel chloride (25–400 *µ*M) for 48 h reduced cell viability in a dose-dependent manner. The apoptotic rates of the 25, 50, 100, 200 and 400 *µ*M treatment groups were 14.4, 31.4, 45.7, 61.9 and 65.7%, respectively. The apoptotic rate of the cells was then examined by Hoechst 33342/PI double staining following the various treatment regimens. In this assay, cytotoxicity of the nickel challenge was indicated as the percentage of dead cells (PI-positive) relative to the total number of cells (Hoechst-positive). The extent of THP-1 cell apoptosis was identified to be proportional to the nickel ion concentration. Incubation of THP-1 for 48 h with low concentrations of nickel (25 and 50 *µ*M) resulted in low apoptotic rates (Fig. 2B and C). By contrast, incubation with high concentrations of nickel ions (>100 *µ*M) was observed to have a negative effect on cell viability as indicated by the increased percentage of dead cells (25.2, 60.7 and 85.7% for 100, 200 and 400 *µ*M nickel, respectively) (Fig. 2D, E and F).

### Differential gene expression of THP-1 cells following treatment with nickel ions

To elucidate the transcriptional events associated with nickel ion-induced THP-1 cytotoxicity, a high-throughput RNA-sequencing assay was conducted using RNA samples extracted from the nickel-challenged cells. Electrophoresis and ethidium bromide staining indicated the successful extraction of RNA products from treated cells. A total of 1 *µ*g total RNA was then pooled from each treatment group (control, 25, 50 and 100 *µ*M NiCl_2_·6H_2_O) for comparison of the gene expression profiles via Illumina sequencing. The differentially expressed genes were then filtered. To characterize the functional consequences of alterations in gene expression associated with nickel cytotoxicity, pathway analysis of DEGs was conducted using the web-based KEGG pathway database. The top four enriched KEGG pathway categories of upregulated genes were cytokine-cytokine receptor interaction, osteoclast differentiation, steroid biosynthesis and the chemokine signaling pathway (Table II). The top four enriched KEGG pathway categories of downregulated genes were olfactory transduction, taste transduction, RNA transport and the mRNA surveillance pathway (Table III).

### Confirmation of cytotoxicity-associated genes

**Figure 3:**
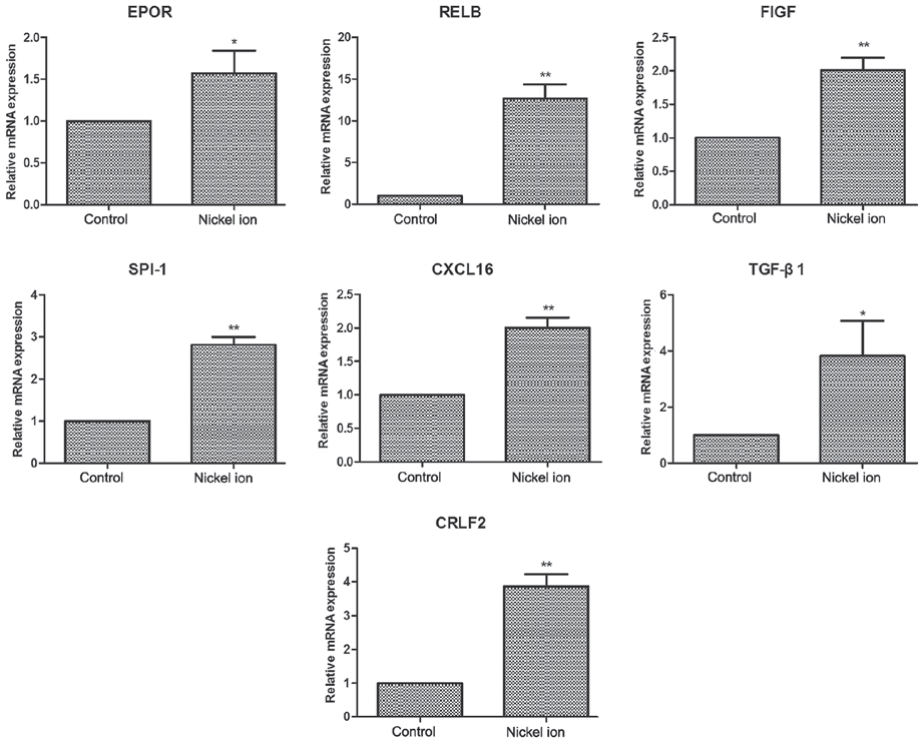
Relative mRNA expression levels of EPOR, RELB, FIGF, SPI-1, CXCL16, TGF-β1 and CRLF2. Values presented in histograms are expressed as the mean ± standard deviation. ^*^P<0.05 and ^**^P<0.01 nickel-treated samples vs. control samples (paired t-test).

The authenticity and reliability of the cytotoxicity-associated genes was then verified by specifically examining the expression levels of ten congenital heart disease-associated genes, including EPOR, As presented in Fig. 3, RELB, FIGF, SPI-1, TGF-β1, CXCL16 and CRLF2 through qPCR analysis. RELB, FIGF, SPI-1, CXCL16 and CRLF2 mRNA expression levels of nickel-treated cells presented were significantly increased compared with those in the control group (P<0.01). TGF-β1 and EPOR mRNA expression levels were also significantly increased following nickel challenge (P<0.05).

## Discussion

The present study investigated the cytotoxic effects of nickel ions on THP-1 cells and the possible mechanisms involved. The results suggested that nickel ions exerted a growth-inhibitory effect on THP-1 cells and in particular, it was demonstrated that the toxicity of nickel ions to THP-1 cells may be associated with the differential expression of certain genes, including EPOR, RELB, FIGF, SPI-1, TGF-β1, CXCL16 and CRLF2.

In the present study, it was observed that the proliferation of THP-1 cells gradually increased following stimulation for a short period of time with low concentrations (25–100 *µ*M) of nickel ions, which may be associated with the incompletely compromised ability of the cells to resist and metabolize the insult. However, with the increases in the nickel ion concentration and incubation time, a rapid decline in the number of live cells was observed. The cells began to die when incubated with high concentrations of nickel ions (200–800 *µ*M). Cell viability analysis suggested that the concentration and incubation time with the nickel ions may be two important indicators of the toxic effect of nickel. Thus, correct assessment of nickel ion toxicity requires to comprehensively take the concentration and time period of its use into account.

Multiple studies have examined the association between nickel exposure and blood and heart diseases (11,12). The toxicity of the nickel ion to the heart has become a focus of research in recent years since nickel-associated products are being increasingly used in heart disease therapy (13). Accumulating evidence has demonstrated that nickel exposure negatively regulates cardiac function (14,15). The results from a previous study indicated that heart rate variability (a predictor for arrhythmias, mortality risk and severity of illness) was regulated by nickel-induced oxidative-inflammatory responses (14). Thus, it is suggested that nickel may cause certain cardio-regulatory responses and may induce varying responses during systolic and diastolic phases (15).

Although the phenomenon of nickel-induced cardiotoxicity has raised increasing concerns, only few studies are available on the mechanism of nickel ion-induced toxicity to the heart during its use in congenital heart disease (16). Previous studies have demonstrated that congenital heart disease is regulated and affected by multiple genes and pathways, including EPOR, SPI-1, p38/MAPK and TGF-β1 (17–20). It remains to be fully elucidated whether there is an association between nickel toxicity and congenital heart disease, and if so, what the associated genes and pathways are. In the present study, the expression levels of EPOR, RELB, FIGF, SPI-1, TGF-β1, CXCL16 and CRLF2 were observed to be significantly increased subsequent to nickel ion stimulation in THP-1 cells. Nickel can also generate cytotoxic reactive oxygen species through TGF-β1 activation (20). CRLF2 gene alterations have been observed to correlate with poor prognosis in Japanese BCR-ABL1-negative high-risk B-cell precursor acute lymphoblastic leukemia (21), which suggests that the downregulation of CRLF2 may block the inflammatory effect of nickel ions. FIGF was detected in adult lung and heart tissues and was observed to exhibit mitogenic activity on fibroblasts (22,23). CXCL16 appears to have a role in the homing of CD4(+) T cells in acute and chronic rejection models of heart allotransplantation (24). Nickel ion stimulation exerted differential effects on these heart disease-associated genes, which may indicate that the toxicity mechanism of the nickel ion may be regulated by these genes. Further studies, including loss-of-function analysis by RNA inference and *in vivo* experiments, are required to fully elucidate the mechanistic significance of these genes and pathways in the observed nickel cytotoxicity.

In conclusion, the present study demonstrated that the toxicity of the nickel ion to THP-1 cells may be controlled by or is associated with certain genes, including EPOR, RELB, FIGF, SPI-1, TGF-β1, CXCL16 and CRLF2. These observations provided insight and advance the understanding of the genetic basis of nickel ion-induced toxicity in its therapeutic use for congenital heart disease.

## Figures and Tables

**Table I:** Primer sequences for quantitative polymerase chain reaction.

Genes	Sequence
EPOR	Forward: GGGCAACTACAGCTTCTCCT
	Reverse: ATGGCATGGACTGTGGTCAT
RELB	Forward: TGATCCACATGGAATCGAGA
	Reverse: CAGGAAGGGATATGGAAGCA
FIGF2	Forward: ATGGACCAGTGAAGCGATCAT
	Reverse: GTTCCTCCAAACTAGAAGCAGC
SPI-1	Forward: GAAAGGTGGGTGAAAGGACCA
	Reverse: TGTTGGACTCCTTTGGGCAG
TGF-β1	Forward: TACAGCACGGTATGCAAGCC
	Reverse: GCAACCGATCTAGCTCACAGAG
CXCL16	Forward: GACATGCTTACTCGGGGATTG
	Reverse: GGACAGTGATCCTACTGGGAG
CRLF2	Forward: AGTGACGGTGACGTGTTCTG
	Reverse: CTATGGTGACGTTGCAGGTATT
GAPDH	Forward: TGTTCGTCATGGGTGTGAAC
	Reverse: ATGGCATGGACTGTGGTCAT

**Table II:** Top four enriched Kyoto Encyclopedia of Genes and Genomes pathway categories of upregulated genes.

Pathway	P-value	Upregulated genes
Cytokine-cytokine receptor interaction	6.77×10^−8^	TGFB1, CXCR4, TNFRSF1B, CD70, CCL5, EGFA, VEGFB, TNFRSF10B, TNF, IL10RA, IL3RA, TNFSF9, CSF2RA, EPOR, TNFSF13B, FLT3LG, CD40, IL2RG, IL23A, CSF1, CXCL16, PDGFA, TNFRSF12A, CRLF2, CCL2, CCL24, CTF1, CCL3, FIGF, CCL13, INHBE, LTA, CCL4, CCL21, XCR1, LEP, TNFSF18, CCL3L3, CL3L1, GDF6, CCL4L2, IL17B, GH1, CXCL11, TNFRSF4, INHBA, CCR7, CCR5, CCL26, IFNB1, GDF5, TNFRSF18, CCL8
Osteoclast differentiation	1.07×10^−4^	SPI1, CYBA, TGFB1, NCF4, JUND, RAC1, STAT1, GAB2, NFKBIA, FCGR1A, JUN, PPARG, IKBKG, LILRB5, TNF, NFKB2, LILRA5, RELB, LILRB2, OSCAR, NCF1, SOCS3, CSF1, SOCS1, FCGR1B, BLNK, LILRB3, LILRA6, IFNB1
Steroid biosynthesis	1.13×10^−4^	FDFT1, SQLE, DHCR7, EBP, SDHL, TM7SF2, HSD17B7, CYP51A1, SOAT2
Chemokine signaling pathway	2.41×10^−4^	GNB2, CXCR4, RAC1, STAT1, FOXO3, CCL5, HCK, NFKBIA, NFKBIB, HRAS, IKBKG, SHC2, FGR, ARRB2, NCF1, ADCY2, CXCL16, AC092535.1, CCL2, GRK7, CCL24, CCL3, CCL13, CCL4, CCL21, CXCR1, ADCY8, CCL3L3, GNGT2, CCL3L1, CCL4L2, CXCL11, CCR7, CCR5, CCL26, CCL8

**Table III:** Top four enriched Kyoto Encyclopedia of Genes and Genomes pathway categories of downregulated genes.

Pathway	P-value	Downregulated genes
Olfactory transduction	2.91×10^−4^	PRKACB, CAMK2D, PDC, CNGA4, CNGB1, OR2V1, CYorf17, OR11L1, OR10A2, OR52L1, OR52K1, OR6V1, OR2T33, DAPL1, OR6K3, OR13G1
Taste transduction	1.22×10^−3^	PRKACB, TAS2R10, TAS2R50, TAS2R4, TAS2R46, GRM4, TAS2R43, TAS2R13, TAS2R60, TAS2R3, GNG13, TAS2R39
RNA transport	1.61×10^−3^	EIF4G2, XPO1, TPR, TMEM48, PNN, NUP205, DDX39B, PRMT5, NUP155, NUP160, NCBP1, POP1, NUP50, GEMIN5, NUP107, NUP88, NUP54, NXT2, NXF3, NUPL1, THOC1, SUMO1, EIF4E, NUP43, TRNT1, RPP40, PABPC1L, SUMO4, PABPC4L, NXF2B
mRNA surveillance pathway	2.13×10^−3^	GSPT1, NUDT21, PNN, PAPOLA, DDX39B, CPSF3, NCBP1, CSTF3, RNGTT, HBS1L, NXT2, NXF3, PAPOLG, CSTF2T, PPP2R3A, PABPC1L, PABPC4L, PAPOLB, NXF2B
